# Comparison of body mass index and waist circumference as predictors of cardiometabolic health in a population of young Canadian adults

**DOI:** 10.1186/1758-5996-2-28

**Published:** 2010-05-12

**Authors:** Darren R Brenner, Kasia Tepylo, Karen M Eny, Leah E Cahill, Ahmed El-Sohemy

**Affiliations:** 1 Department of Nutritional Sciences, University of Toronto, Toronto, Ontario, Canada; 2 The Dalla Lana School of Public Health, University of Toronto, Toronto, Ontario, Canada

## Abstract

**Background:**

This study aimed to investigate whether waist circumference (WC) or body mass index (BMI) is a better predictor of blood lipid concentrations among young men and women from different ethnocultural groups.

**Methods:**

Participants were 1181 healthy men (n = 358) and women (n = 823) aged 20-29 years taken from the cross-sectional Toronto Nutrigenomics and Health Study. Analyses were conducted separately for men and women, and for Caucasian and East Asian ethnocultural groups. Serum triglycerides (TG) and total to HDL cholesterol ratio (TC:HDL cholesterol) were used as outcomes. Associations between the adiposity and blood lipid measures were examined using partial correlations and odds ratios derived from logistic regression models.

**Results:**

WC had a stronger association with serum lipid concentrations than BMI. WC was significantly related to TG and TC:HDL cholesterol after adjusting for BMI and covariates among men and women (P ≤ 0.01). However, after adjusting for WC and covariates, BMI was not significantly associated with the two serum lipid measures. WC was a better predictor of TG and TC:HDL among all sex and ethnocultural subgroups except among East Asian women where little difference between the two measures was observed.

**Conclusions:**

WC is a stronger predictor of cardiometabolic health when compared with BMI among young adults, especially among men.

## Background

Excess body fatness is a risk factor associated with premature mortality [1,2], type 2 diabetes [3,4] and cardiovascular disease (CVD) [1,5]. The high prevalence of overweight and obesity among young adults [6] has raised concern about the increased risk of CVD in younger individuals [7]. In order to develop appropriate preventative strategies, there is a need to understand the association between measures of adiposity and cardiometabolic risk factors such as blood levels of cholesterol [8] and triglycerides [9,10] in young populations. A systematic examination of the evidence for the relationship between serum cholesterol and heart disease shows that serum cholesterol reduction in populations with high rates of heart disease is an effective and safe method of reducing heart disease rates [11]. For example, a 10 mg/dl rise in LDL-cholesterol has been associated with an approximately 10 percent increased risk of heart disease over a period of 5 to 10 years among [12]. However, additional investigation among young populations with LDL levels in the normal range is required to determine whether increases within this range continue to affect risk. Meta-analyses of population-based prospective studies have concluded that serum triglyceride levels are a risk factor for CVD, independent of cholesterol levels, for both men and women in the general population [13,14].

Body mass index (BMI) is a commonly used indicator of obesity and has been associated with an unfavourable lipid profile consisting of elevated triglycerides, total cholesterol, and low density lipoprotein (LDL) cholesterol and low high density lipoprotein (HDL) cholesterol in men and women as young as 20 years of age [15,16]. BMI is often used in clinical settings to estimate body fat and to assess risk among adults [17]. The use of BMI, however, has limitations because it has been shown that current BMI cutoffs may underestimate obesity and the associated health risk factors among populations that are not Caucasian [18]. Furthermore, it does not account for factors such as body size [19] and body fat distribution such as abdominal obesity [20]. There is growing evidence to support an association between abdominal fat and CVD incidence [21,22] and outcomes such as cardiometabolic risk factors [23-25]. Waist circumference (WC), a simple measure of abdominal fat, has been observed to be a stronger predictor of obesity-related risk factors than BMI in older adults [23-28]. Studies examining the relative strengths of WC and BMI as predictors of cardiometabolic risk in young adults from different ethnocultural backgrounds are limited. The objective of this study was to compare the association of WC and BMI with serum lipid concentrations among young men and women between the ages of 20-29 from an ethnoculturally diverse population.

## Methods

### Study population

Participants were from the Toronto Nutrigenomics and Health Study, which is a cross sectional study of healthy men and women aged 20-29 years recruited from the University of Toronto campus. The total sample for this analysis included 1181 men (n = 358) and women (n = 823) recruited between September 2004 and March 2009. The sample sizes for the subgroups consisted of 566 Caucasian men (n = 170) and women (n = 396) and 406 East Asian men (n = 112) and women (n = 294). The study protocol was approved by the Research Ethics Board at the University of Toronto, and all subjects provided written informed consent.

### Blood lipids

The outcome measures used for this analysis were serum triglycerides (TG) and total to HDL-cholesterol ratio. After a minimum 12-hour overnight fast, blood samples were collected at LifeLabs medical laboratory services (Toronto, Ontario, Canada) for blood lipid measurements using standard laboratory procedures and controls [29]. Total cholesterol, HDL-cholesterol and triglycerides were measured using a chromatographic enzymatic method with a Siemens Advia^® ^2400 analyzer (Siemens Healthcare Diagnostics, Deerfield, Illinois, USA). LDL-cholesterol was calculated using the Friedewald formula for samples with triglyceride values between 0.30 and 4.52 mmol/L [30]. Total to HDL cholesterol ratio (TC:HDL cholesterol) was calculated as total cholesterol divided by HDL cholesterol. No subjects reported taking lipid lowering medications.

### Adiposity measurements

BMI and WC were the independent predictors used in the analysis. Anthropometric measurements were taken by trained personnel with the subject dressed in light clothing with shoes removed. WC was measured to the nearest 0.1 centimeter at the mid-point between the lower ribs and the iliac crest. Height was measured to the nearest 0.1 centimeter with a wall-mounted stadiometer (model Seca 206, Seca Corporation, Hanover, MD, USA). Body weight was measured to the nearest 0.1 kilogram using a digital scale. BMI (kg/m^2^) was calculated using the height and weight measurements. Waist circumference was measured twice to the nearest 0.1 cm by placing a non-stretchable measuring tape at the level of the smallest area of the waist. Participants were asked to keep their arms to their sides and breathe normally as measurements were taken. A third measurement was taken if the difference between the first two was ≥ 1 cm. The mean of the waist circumference measurements was calculated.

### Adjustment variables

Ethnocultural group, smoking status, and physical activity were covariates included in the analysis. Ethnocultural status was self-reported and classified into four groups: Caucasian, East Asian, South Asian or other, which includes individuals who reported being from two or more ethnocultural groups. Current smokers were defined as those who smoked at least one cigarette per day for 1 month or longer. Modifiable physical activity was measured by questionnaire and expressed as modifiable metabolic equivalent (MET)-hours per week, which represents both leisure and occupational activity, but not including sedentary hours of sleeping or sitting. One MET is equal to 1 kcal expended per kg body weight per hour sitting at rest [31].

### Statistical methods

All analyses were conducted with SAS software (SAS version 9.1, SAS Institute, Cary, NC). Analyses were carried out separately for men and women since men and women differ in their cardiometabolic risk profiles [32]. Subject characteristics for men and women are presented as mean ± standard deviation for all continuous variables and were compared using unpaired t-tests with unequal variances for normally distributed variables and the Wilcoxon rank sum test was used for skewed variables. Categorical variables were compared with a chi-square test. Two methods were used to determine whether WC or BMI had a stronger association with TC:HDL cholesterol and TG, including partial correlation analysis and logistic regression. First, we computed partial Spearman correlation coefficients between: (1) WC and the outcome variables after adjusting for BMI, age, ethnocultural group, smoking status and physical activity, and (2) BMI and the dependent variables after adjusting for WC, age, ethnocultural group, smoking status and physical activity. The coefficients and the associated *P *values were used to assess whether one was a better predictor of risk than the other. In the second approach, TC:HDL cholesterol and TG were divided into quartiles, and the highest quartile was designated as the high risk group, with the bottom three quartiles being grouped and serving as the reference group. Logistic regression was used to estimate the multivariate-adjusted odds ratios and 95% confidence intervals (CI) of high TC:HDL cholesterol and TG associated with one standard deviation increase in WC and BMI, adjusted for age, ethnocultural group, smoking status, and physical activity. A comparison of a change in one standard deviation was used in order to provide equal comparisons between the measures. The Subgroup analyses were conducted for Caucasian and East Asian men and women using partial correlations and odds ratios as described above. In order to compare predictive ability of BMI and WC, the c-statistic was used [33]. The α error was set at .05 and all reported *P *values are 2-sided.

## Results

Subject characteristics for men and women are provided in Table 1. Women had a significantly lower BMI (P < 0.001), lower WC (P < 0.001) and lower TC:HDL cholesterol (P < 0.001) compared to men. The sample consisted of predominantly Caucasian and East Asian participants (79% for men, 84% for women). Among males WC was significantly higher among Caucasians than East Asians (P < 0.001), while no differences were observed for BMI or TC:HDL cholesterol and TG. Among females WC and BMI were significantly higher among Caucasians than East Asians (P < 0.001), while no differences were observed for TC:HDL cholesterol and TG.

**Table 1:** Subject characteristics for men and women (n = 1181)

	Men (n = 358)	Women (n = 823)	*P *value
Age (years)	22.8 ± 2.4	22.5 ± 2.4	0.08
Height (cm)	175.9 ± 7.2	163.2 ± 6.5	< 0.001
Weight (kg)	73.1 ± 12.7	59.7 ± 10.7	< 0.001
Body mass index (kg/m^2^)	23.6 ± 3.5	22.4 ± 3.5	< 0.001
Waist circumference (cm)	80.1 ± 9.1	71.3 ± 7.6	< 0.001
Total cholesterol (mmol/L)	4.0 ± 0.7	4.3 ± 0.8	< 0.001
LDL-cholesterol (mmol/L)	2.2 ± 0.6	2.2 ± 0.6	0.72
HDL-cholesterol (mmol/L)	1.3 ± 0.3	1.7 ± 0.4	< 0.001
Total cholesterol to HDL ratio	3.2 ± 0.9	2.7 ± 0.6	< 0.001
Triglycerides (mmol/L)	1.0 ± 0.6	1.0 ± 0.5	0.80
Smokers (%)	10%	5%	0.0023
Modifiable METS	7.9 ± 3.3	7.6 ± 3.01	0.17
Ethnocultural group (%)			
Caucasian	48%	48%	0.84
East Asian	31%	36%	0.14
South Asian	14%	9%	0.005
Other	7%	8%	0.87

Values shown as mean ± standard deviation for continuous variables. Categorical variables are presented as percentages. *P *values from Wilcoxon test shown for variables age and TG. Unpaired t-test with unequal variances was used for all other continuous variables. Chi-square test used for categorical variables.

Spearman correlations for BMI and WC with blood lipid measures are shown in Table 2. WC and BMI were strongly associated for men (r = 0.88) and women (r = 0.85). Both measures of adiposity were significantly correlated with blood lipids, and correlations were higher for men than for women. Among men, WC correlated better than BMI with the two outcome measures, whereas among women there was no apparent difference between the two measures when examining raw correlations. Results of partial correlation analyses using Spearman coefficients are given in Table 3. After adjusting for BMI and other covariates, there was a significant association between WC and the two outcome measures. However, after adjusting for WC and other covariates, the association between BMI and the two dependent variables was no longer significant.

**Table 2:** Spearman correlations of WC and BMI with blood lipids (n = 1181)

	Men		Women	
		
	BMI	WC	BMI	WC
BMI	-	0.88**	-	0.85**
WC	0.88**	-	0.85**	-
Total cholesterol	0.18*	0.17*	0.10	0.09
LDL-cholesterol	0.25**	0.25**	0.12*	0.12*
HDL-cholesterol	-0.27**	-0.32**	-0.20**	-0.22**
TC:HDL cholesterol	0.35**	0.38**	0.26**	0.26**
TG	0.21**	0.24**	0.16**	0.20**

* *P *< 0.05, ** *P *< 0.0001

WC- Waist circumference (cm)

TG - Triglycerides (mmol/L).

**Table 3:** Adjusted Spearman partial correlations (n = 1181)

	Men		Women	
		
	TG	TC:HDL cholesterol	TG	TC:HDL cholesterol
WC^a^	r = 0.14 *P *= 0.007	r = 0.20 *P *< 0.001	r = 0.12 *P *= 0.0008	r = 0.09 *P *= 0.01
BMI^b^	r = -0.024 *P *= 0.66	r = 0.0004 *P *= 0.99	r = 0.01 *P *= 0.78	r = 0.07 *P *= 0.04

^a ^Adjusted for BMI, age, ethnocultural group, smoking status, physical activity.

^b ^Adjusted for WC, age, ethnocultural group, smoking status, physical activity.

WC - Waist circumference (cm)

TG - Triglycerides (mmol/L).

To examine the association of WC and BMI as continuous variables with high TC:HDL cholesterol and TG, both adiposity measures were standardized separately for men and women, and logistic regression was used to estimate the odds ratios (95% CI) (Table 4). In all cases, the odds ratio for high TG and TC:HDL cholesterol associated with a one standard deviation increase in WC were higher than when compared to a one standard deviation increase in BMI. This was most apparent among men, particularly for high TC:HDL cholesterol (OR 2.45, (95% CI 1.81-3.33) for WC versus OR 1.99, (95% CI 1.51-2.63) for BMI). Among women, the odds ratios associated with a one standard deviation increase in WC were only slightly higher when compared to a one standard deviation increase in BMI. The c-statistic was higher for WC than for BMI in all gender-stratified models.

**Table 4:** Adjusted^a ^odds ratios (95% confidence interval) and concordance (c) statistics^b ^for high TC:HDL cholesterol and TG associated with 1 standard deviation of BMI and WC^c ^(n = 1181)

	Men	Women
High TG^d^		
BMI	1.65 (1.27-2.13) 0.685	1.43 (1.22-1.68) 0.619
WC	1.75 (1.33-2.30) 0.695	1.57 (1.34-1.85) 0.639
High TC:HDL cholesterol^c^		
BMI	1.99 (1.51-2.63) 0.707	2.16 (1.80-2.59) 0.697
WC	2.45 (1.81-3.33) 0.734	2.21 (1.84-2.67) 0.700

^a ^Adjusted for age, ethnocultural group, smoking status, and physical activity level.

^b ^Concordance statistics from identical models for BMI and WC

^c ^WC and BMI treated as standardized continuous variables.

^d ^High TG and TC:HDL cholesterol defined as the highest quartile, with quartiles 1-3 serving as reference group.

WC - Waist circumference (cm)

TG - Triglycerides (mmol/L).

The association between blood lipids and the two measures of adiposity were examined separately for Caucasian and East Asian men and women. Results from the partial correlation analyses are summarized in Table 5. After adjusting for WC, BMI was not significantly associated with TC:HDL cholesterol and TG. After adjusting for BMI, WC was significantly associated with the two blood lipid measures for Caucasian and East Asian men (*P *< 0.05). WC was associated with TC:HDL cholesterol after adjusting for BMI (r = 0.12, P < 0.05) among Caucasian women. The multivariate adjusted odds ratios (95% CI) for high TC:HDL cholesterol and TG associated with a one standard deviation increase in BMI and WC are given in Table 6. Compared to BMI, the odds ratios for WC were higher for Caucasian and East Asian men, particularly for high TC:HDL cholesterol. Among Caucasian women, a one standard deviation increase in WC was associated with a higher odds ratio for high TC:HDL cholesterol and TG when compared to a one standard deviation increase in BMI. Among East Asian women a one standard deviation increase in BMI was associated with a higher odds ratios for TC:HDL cholesterol. The c-statistic was also higher for WC than BMI for all subgroups with the exception of Asian women.

**Table 5:** Adjusted Spearman partial correlations for Caucasian and East Asian subgroups

	Men		Women	
		
	TC:HDL cholesterol	TG	TC:HDL cholesterol	TG
Caucasians				
BMI^a^	-0.11	-0.09	0.04	0.02
WC^b^	0.27*	0.22*	0.12*	0.06
East Asians				
BMI^a^	0.01	-0.11	0.12*	0.05
WC^b^	0.23*	0.22*	0.01	0.07

^a ^Adjusted for WC, age, smoking status, physical activity.

^b ^Adjusted for BMI, age, smoking status, physical activity.

* *P *< 0.05

WC - Waist circumference (cm)

TG - Triglycerides (mmol/L).

**Table 6:** Adjusted^a ^odds ratios (95% confidence interval) and concordance (c) statistics^b ^for high TC:HDL cholesterol and TG^c ^associated with 1 standard deviation of BMI and WC^d ^for Caucasian and East Asian subgroups

	Men		Women	
		
	High TC:HDL cholesterol	High TG	High TC:HDL cholesterol	High TG
Caucasians				
BMI	1.60 (1.12, 2.28) 0.669	1.86 (1.29, 2.69) 0.679	1.99 (1.56, 2.54) 0.683	1.20 (0.96, 1.49) 0.591
WC	2.06 (1.41, 3.01) 0.717	2.30 (1.54, 3.42) 0.708	2.12 (1.65, 2.75) 0.705	1.28 (1.03, 1.59) 0.597
East Asians				
BMI	2.77 (1.63, 4.71) 0.763	1.78 (1.12, 2.83) 0.719	1.63 (1.14, 2.15) 0.647	1.51 (1.15, 1.98) 0.614
WC	2.88 (1.69, 4.89) 0.782	1.71 (1.09, 2.70) 0.724	1.53 (1.17, 2.00) 0.639	1.50 (1.14, 1.96) 0.609

^a ^Adjusted for age, smoking status, and physical activity level.

^b ^Concordance statistics from identical models for BMI and WC

^c ^High TC:HDL cholesterol and TG defined as the highest quartile, with quartiles 1-3 serving as reference group.

^d ^WC and BMI treated as standardized continuous variables.

WC - Waist circumference (cm)

TG - Triglycerides (mmol/L).

## Discussion

We observed that WC was better than BMI at predicting serum lipid levels in a population of young adults from different ethnocultural groups. Both WC and BMI were significantly associated with TG and TC:HDL cholesterol, however, only WC remained significant after adjusting for BMI and other covariates.

Consistent with previous studies [24,25], BMI and WC were positively correlated. Also in concordance with other investigations [4], WC remained a significant predictor of CVD risk factors, independently of BMI. As has been previously reported [15,16], crude BMI was inversely associated with total cholesterol and triglycerides, and inversely associated with HDL in the present study. However, WC and not BMI had the stronger association with TG and TC:HDL cholesterol after adjusting for covariates. Several other studies in adults have reported a stronger positive association between CVD risk factors such as TG and TC:HDL cholesterol with abdominal adiposity measured by either waist circumference or waist-to-hip ratio, than with overall adiposity as measured by BMI [24-26]. These studies investigated adult subjects of a broad age range and were not specific to young adults, a target age ideal for the prevention of CVD. To our knowledge, the present study among the first to compare WC and BMI as predictors of cardiometabolic health in young adults from different ethnocultural backgrounds.

In the current analysis of young adults, the strength of the association between the measures of adiposity and blood lipids varied by both sex and ethnocultural group. WC was observed to be the more useful measure in predicting blood lipids for both sexes, especially among Caucasian men. The strength of WC over BMI was not observed when the analysis was restricted to Caucasian and East Asian women. When comparing different measures of adiposity, among a young East Asian population, previous studies have reported that BMI and WC were useful predictors of CVD risk factors, including blood lipids, for men [34]. Similarly in a cross-sectional study of female monozygotic Asian twins, BMI performed as good as or better than WC in predicting lipid risk factors including TG and TC:HDL cholesterol [35]. The observations supporting interchangeable use of BMI and WC among East Asian subjects may be due to the ability of both measures to predict trunk obesity as measured by DEXA in young Asian men and women [36]. The differences in the strength of the association observed in men versus women could be due to higher muscle mass in men [37].

Findings of the present study provide further evidence that abdominal fat is a stronger predictor of blood lipids than overall body size, as measured by BMI, which is the most widely used measure of body size because of its practical value in both clinical practice and large-scale epidemiological studies to define obesity and overweight (ie. An individual with a BMI > 25 is classified as overweight, and > 30 is classified as obese). However, a number of limitations with using BMI have been recognized [37], including the inability to account for the wide variation in body fat distribution [20], and failure to distinguish between the respective contributions of fat and muscle to body weight [20,37]. Since abdominal adiposity can vary greatly at a given BMI [20], excess weight measures that capture the increased health risk due to abdominal fat have an advantage when compared to BMI. The underlying basis may be that WC has a stronger relationship with adipose tissue distribution than BMI [26], making WC a better anthropometric marker most associated with the adverse blood lipid profile that accompanies excess adiposity. Our observation is consistent with a large body of evidence implicating abdominal obesity in the pathogenesis of numerous metabolic diseases [23,26]. Although the mechanisms that explain the increased health risk predicted by WC are not firmly established, it is often suggested that the added risk is explained by the metabolic complications associated with elevations in abdominal obesity, such as increased lipogenesis and lipolysis of abdominal fat resulting in hyperlipidemia [38]. However, there may be other mechanisms involved since BMI and WC were reported to independently contribute to the prediction of non-abdominal, abdominal subcutaneous and visceral fat in white men and women [39], demonstrating the potential importance of using both BMI and WC in clinical practice.

The present study is strengthened by the use of data from an ethnoculturally diverse sample of young adults, a population that had yet been fully examined in comparing central and overall adiposity measures for CVD risk characterization. Analyses were carried out for the entire sample, and separately for Caucasian and East Asian groups to observe potential ethnic-specific effects. The categorization and standardization of BMI and WC in logistic regression analysis allowed for the direct comparison of the odds ratios for these two measures. WC consistently produced higher odds ratios and significant partial correlation coefficients with high blood lipid levels compared to BMI. Simultaneous adjustment for age, ethnocultural group, smoking status and physical activity in the regression models allowed for control of any potentially confounding effects of these factors.

Several potential limitations should be considered in interpreting the results of the present study. Cross-sectional data was used and as such only predictive models of intermediate cardiometabolic measures could be examined. Future use of longitudinal designs would provide stronger evidence for the relative abilities of WC and BMI in risk prediction. It should also be noted that the majority of the population falls within the healthy ranges of usual clinical distributions of cholesterol levels and measures of adiposity and that the results may not be fully extrapolated to a more clinically advanced population with higher levels. Another limitation of the present study is that stratification for ethnocultural groups other than Caucasian and East Asian subgroups was not conducted due to the smaller sample sizes. Finally, only single measurements were used, however, a recent investigation of the reliability and stability of plasma lipid biomarkers including TG and cholesterol concluded that measurements of lipid biomarkers from a single spot blood sample are a good representation of the average blood levels of these biomarkers [40]. In recent years, the reliability of blood lipid measurement has become generally high as evidenced by a recent study reporting a coefficient of variance less than 2% between repeat samples [41]. We used standardized laboratory services employed by practicing clinicians with high measures of quality control. The high-quality standardization of the anthropometric measurements in the present study reduced measurement error and potential bias.

There are several implications of the findings from the present study. The analyses show the potential value of assessing WC as an indicator of obesity-associated health risk in young men and women. WC may, therefore, have a higher utility for clinical prediction of disease risk. Our findings support the growing evidence that WC can serve as a practical and non-invasive screening method for the lipid profile risks that often accompany overweight and obesity. Preventative programs to reduce CVD risk by improving lipid levels in young adults should include more emphasis on reducing central adiposity and maintaining ideal waist circumference.

## Conclusion

In conclusion, in this cross-sectional study of Canadian men and women aged 20-29 years, WC is a better predictor of serum concentrations of TG and TC:HDL cholesterol than is BMI. This observation was most apparent among men and when restricted to Caucasian and East Asian subgroups. The implications of these findings underscore the importance of WC, independent of BMI, as a marker of cardiometabolic health in young adults.

## Abbreviations

BMI: Body Mass Index; CVD: Cardiovascular disease; DEXA: Dual energy X-ray absorptiometry; HDL: High density lipoprotein; LDL: low density lipoprotein; MET: Metabolic equivalent; TG: Serum triglycerides; TC:HDL: cholesterol: total to HDL cholesterol ratio; WC: Waist Circumference.

## Competing interests

The authors declare that they have no competing interests.

## Authors' contributions

DB & KT conducted the analyses and drafted the manuscript. KE & LC were involved in drafting the manuscript and provided critical revisions. AE conceived of the study design, coordination, was responsible for data collection, interpretation of data and helped to draft the manuscript. All authors read and approved the final manuscript.
